# Balanced Excitatory and Inhibitory Synaptic Currents Promote Efficient Coding and Metabolic Efficiency

**DOI:** 10.1371/journal.pcbi.1003263

**Published:** 2013-10-03

**Authors:** Biswa Sengupta, Simon B. Laughlin, Jeremy E. Niven

**Affiliations:** 1 Wellcome Trust Centre for Neuroimaging, University College London, London, United Kingdom; 2 Centre for Neuroscience, Indian Institute of Science, Bangalore, India; 3 Department of Zoology, University of Cambridge, Cambridge, United Kingdom; 4 School of Life Sciences and Centre for Computational Neuroscience and Robotics, University of Sussex, Falmer, United Kingdom; University of California San Diego, United States of America

## Abstract

A balance between excitatory and inhibitory synaptic currents is thought to be important for several aspects of information processing in cortical neurons *in vivo*, including gain control, bandwidth and receptive field structure. These factors will affect the firing rate of cortical neurons and their reliability, with consequences for their information coding and energy consumption. Yet how balanced synaptic currents contribute to the coding efficiency and energy efficiency of cortical neurons remains unclear. We used single compartment computational models with stochastic voltage-gated ion channels to determine whether synaptic regimes that produce balanced excitatory and inhibitory currents have specific advantages over other input regimes. Specifically, we compared models with only excitatory synaptic inputs to those with equal excitatory and inhibitory conductances, and stronger inhibitory than excitatory conductances (i.e. approximately balanced synaptic currents). Using these models, we show that balanced synaptic currents evoke fewer spikes per second than excitatory inputs alone or equal excitatory and inhibitory conductances. However, spikes evoked by balanced synaptic inputs are more informative (bits/spike), so that spike trains evoked by all three regimes have similar information rates (bits/s). Consequently, because spikes dominate the energy consumption of our computational models, approximately balanced synaptic currents are also more energy efficient than other synaptic regimes. Thus, by producing fewer, more informative spikes approximately balanced synaptic currents in cortical neurons can promote both coding efficiency and energy efficiency.

## Author Summary

The adult human brain consumes more than 20% of the resting metabolism. With ∼19–23 billion neurons, the cerebral cortex consumes much of this energy, mainly to restore ion gradients across membranes for electrical signaling. Even small increases in the average spike rate of cortical neurons could cause the cortex to exceed the energy available for the whole brain. Consequently, the cortex is likely to be under considerable selective pressure to reduce spike rates but, given its important roles in behavior, to maintain information processing. Numerous experimental studies have shown that excitatory and inhibitory synaptic currents are balanced in cortical neurons. Could this feature of cortical neurons contribute to their efficiency? We tested this by making comparisons among computational models with different amounts of inhibition and excitation: excitation only, equal excitation and inhibition (balanced synaptic conductances), and more inhibition than excitation (balanced synaptic currents). Our simulations show that computational models with balanced synaptic currents have similar information rates to the other regimes but achieve this with fewer, more informative spikes that consume less energy. Therefore, in comparison to other synaptic regimes, balanced synaptic currents have the highest coding efficiency and the highest energy efficiency.

## Introduction

Cortical neurons receive many thousands of weak (sub-millivolt) excitatory synaptic inputs [1], the majority of which originate from other local or distant neurons within the cortex [2], [3]. The currents generated by these excitatory inputs are approximately balanced by inhibitory currents [4], [5] generated by fewer, stronger synaptic inputs from inhibitory interneurons [6]. During ongoing activity *in vivo*, excitatory and inhibitory currents depolarize the membrane from the resting potential to around −60 mV, slightly below the threshold for spike initiation [7]. For excitatory and inhibitory currents to balance at approximately −60 mV, the inhibitory conductances must be larger than excitatory conductances. Operating this close to threshold, small fluctuations in synaptic inputs can depolarize the neuron sufficiently to trigger spikes, giving rise to highly variable interspike intervals, similar to those expected from a Poisson process [4], [5]. Depolarizing the membrane with balanced synaptic currents also reduces the membrane time constant, thereby increasing temporal resolution and extending bandwidth [8]–[10], and alters both the neuron's sensitivity and its working point by changing gain [11]–[14]. Thus, depolarization by balanced excitatory and inhibitory currents affects numerous aspects of information processing in cortical neurons.

Cortical information processing accounts for a considerable proportion of the mammalian brain's energy consumption, and cortical energy usage is dominated by synaptic transmission and action potentials [15]–[17]. The cortex's restricted energy budget places limits on the mean spike rate and hence neural processing, suggesting that the cortex may be under strong selective pressure to save energy and increase efficiency [15], [18]. Balanced synaptic currents increase energy consumption by depolarizing the membrane potential and lowering the input resistance. Consequently, balanced synaptic currents will affect cortical information processing and energy consumption, yet how they do so remains unclear.

To assess the impact of balanced synaptic currents on information coding and energy consumption, we compared single-compartment models with stochastic voltage-gated Na^+^ and K^+^ channels driven by one of three synaptic input regimes; excitatory inputs only, equal excitatory and inhibitory conductances (balanced synaptic conductances), and stronger inhibitory than excitatory conductances (balanced synaptic currents). By quantifying the performance of these models over a range of synaptic input statistics, we show that balanced inhibitory and excitatory synaptic currents increase both coding efficiency (bits/spike) and energy efficiency (ATP molecules/bit) in comparison to the other synaptic input regimes. Two factors contributed to the superior efficiency of models with balanced synaptic currents, their firing rates were lower and their spikes more precise. Thus, our models show that balanced synaptic inputs can improve both the coding efficiency (bits per spike) and the energy efficiency (bits per ATP molecule) of cortical neurons.

## Results

### Single compartment models

**Figure 1:**
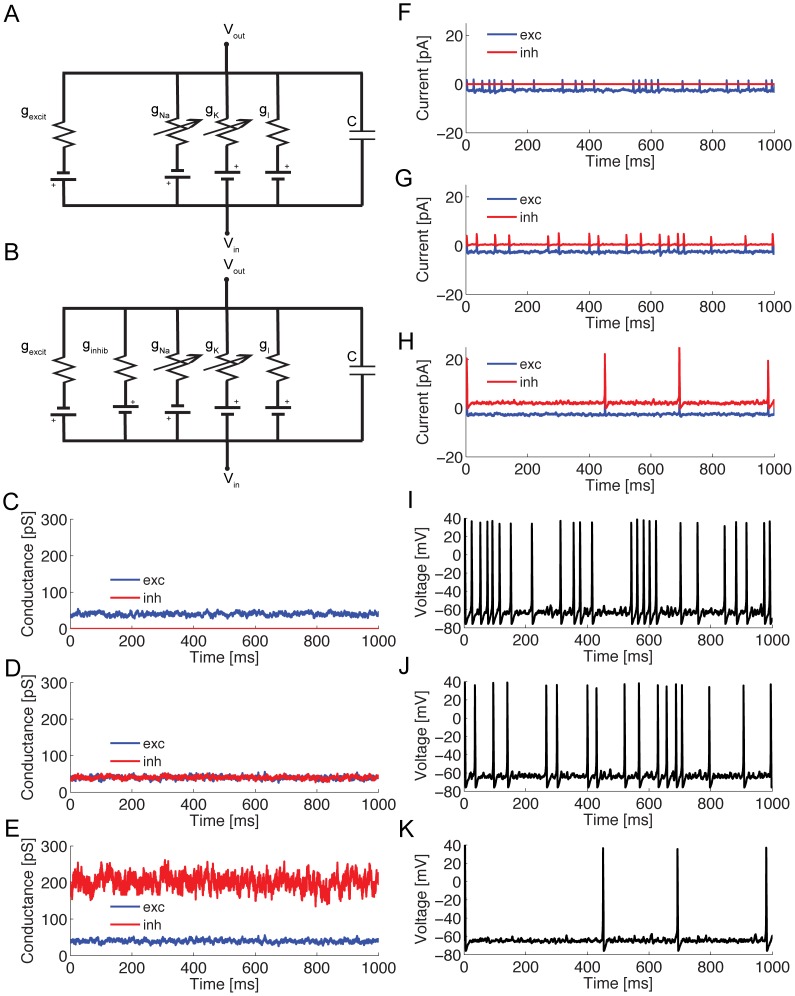
The single compartment model. (A) A circuit diagram of a single compartment model with two voltage-gated conductances, g_Na_ and g_K_, and a leak conductance, g_l_. The model has a capacitance, C, determined by the size of the compartment and receives excitatory synaptic inputs, g_excit_. (B) A circuit diagram of a single compartment model as in A with an additional inhibitory synaptic input, g_inhib_. (C) An example of a low mean, low contrast excitatory conductance waveform. (D) An example of a low mean, low contrast excitatory conductance waveform and an identical inhibitory conductance waveform. (E) An example of a low mean, low contrast excitatory conductance waveform and an inhibitory conductance waveform with five-fold greater mean and standard deviation. (F) The synaptic current evoked by the stimulus shown in C. (G) The synaptic current evoked by the stimulus shown in D. (H) The synaptic current evoked by the stimulus shown in E. (I) The spike train evoked by the stimulus shown in C. (J) The spike train evoked by the stimulus shown in D. (K) The spike train evoked by the stimulus shown in E.

We simulated the responses of a 100 µm^2^ single compartment model containing stochastic voltage-gated Na^+^, K^+^ channels and a non-probabilistic leak conductance, to excitatory synaptic inputs alone (Figure 1A) or to combinations of excitatory and inhibitory inputs (Figure 1B). In the limit of large numbers of Poisson synaptic events with small unitary conductances converging on the post-synaptic compartment, the conductances become a Gaussian white noise process (“the diffusion approximation”) [19]. For synaptic events with a finite time constant, fluctuations in conductance are represented as an Ornstein-Uhlenbeck (OU) process (see Methods) [20], parameterized by the means (*μ_e_, μ_i_*), the standard deviations (*σ_e_, σ_i_*), and the time constant of the excitatory and inhibitory synaptic events (τ*_e_*, τ*_I_* were both fixed at 3.3 ms) [20]. The input conductance contrast is the ratio of *σ* to *μ*. The mean synaptic conductance depends upon the rate, the unitary synaptic event amplitude, and the exponential decay time constant of synaptic events (Eq. 5), whilst the contrast is a function of the rate and the decay time constant (Eq. 7). Therefore, when we increase the conductance contrast we are reducing the frequency with which afferent spikes activate synapses, and when we increase the mean conductance at constant contrast we are increasing event amplitude at constant rate.

We modeled three synaptic input regimes. The first was excitation only (Figure 1C,F,I). In the second regime, balanced conductance, the means and standard deviations of the excitatory and inhibitory synaptic conductances were equal, (Figure 1D,G,J). In the third regime, approximately balanced current, the mean excitatory and inhibitory conductances were adjusted (Figure 1E,H,K) to produce approximately equal inward and outward currents at the mean sub-threshold membrane potential of approximately −64 mV. In this balanced current regime, *μ_i_ = 5μ_e_* and, because inhibitory and excitatory conductances always had the same contrast, *σ_i_* = 5*σ_e_* [7]. All three synaptic regimes evoked action potentials (APs, spikes), the rate of which depended upon the specific regime, and the stimulus mean and contrast (Figure 1). As expected, increasing the inhibitory input reduced spike rates (Figure 1I–K).

### Spike rates

**Figure 2:**
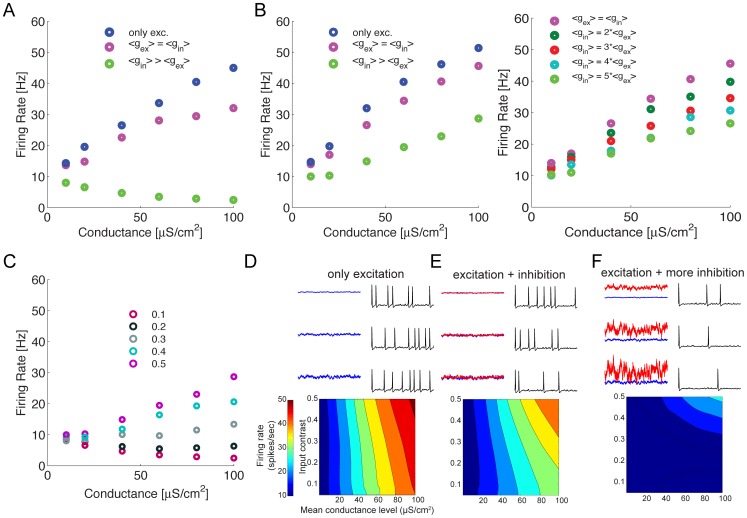
Firing rates of spike trains evoked by three different synaptic input regimes. (A) The firing rate of the single compartment model for three different synaptic regimes; excitation alone, identical excitation and inhibition, and five-fold greater inhibition than excitation. All stimuli have a low contrast (0.1). (B) (left panel)The firing rate of the single compartment model with increasing inhibition. The amount of inhibition varies from none (excitation alone) to five-fold greater inhibition than excitation. All stimuli have a high contrast (0.5). (right panel) The firing rate of the single compartment model with different levels of inhibition. (C) The firing rate of the single compartment model for five-fold greater inhibition than excitation. The stimulus contrast ranges from 0.1 to 0.5. (D) (top panel) Action potentials in response to a mean excitatory (blue trace) conductance of 20 µS/cm^2^ at three different contrasts (top: 0.1, middle: 0.25. bottom: 0.4). (bottom panel) The information rates of spike trains generated by excitatory conductances alone. (E) (top panel) Action potentials in response to a mean excitatory (blue trace) and inhibitory (red trace) conductance of 20 µS/cm^2^ at three different contrasts (top: 0.1, middle: 0.25. bottom: 0.4). The x- and y- scales are identical to that in D. (bottom panel) As in D, except that an identical inhibitory synaptic input has been added. (F) (top panel) Action potentials in response to a mean excitatory (blue trace) conductance of 20 µS/cm^2^ at three different contrasts (top: 0.1, middle: 0.25. bottom: 0.4). The mean and standard deviation of the inhibitory conductance (red trace) is set at five times that of the excitatory conductance. The x- and y- scales are identical to that in D. (bottom panel) As in D, except that the excitatory input is accompanied by a five-fold greater inhibitory synaptic input. The x- and y-axes represent the mean and contrast of the excitatory conductance.

We quantified the differences in the spike rates of the models driven by different synaptic input regimes. Within each regime we varied the means of the excitatory and inhibitory inputs at different contrasts (Figure 2), while keeping *μ_i_ = μ_e_* in the balanced conductance regime, and *μ_i_ = 5μ_e_* in the approximately balanced current regime. At low contrasts (i.e. high synaptic event rates), increasing the mean synaptic conductance in the excitatory regime increases the spike rate from ∼10 spikes/s with minimal input to over 40 spikes/s with 100 μS/cm^2^ (Figure 2A,D). Adding an inhibitory conductance with the same mean conductance so that model operates in the balanced conductance regime, shifts the curve relating mean synaptic conductance to spike rate down, reducing the maximum spike rate to 30 spikes/s with 100 μS/cm^2^ (Figure 2A,E). This downward shift reduces sensitivity, yet increases the range over which the compartment can operate. In the approximately balanced current regime, *μ_i_ = 5μ_e_*, increasing the total mean conductance inverts the trend seen in the other two regimes; spike rates decrease from ∼10 spikes/s to ∼2 spikes/s (Figure 2A,F).

Next we examined responses to higher contrasts that are produced by larger synaptic events occurring at lower rates. In the excitatory regime the spike rate increases with the mean synaptic conductance, from ∼10 spikes/s with no input to ∼50 spikes/s with 100 μS/cm^2^ (Figure 2B,D). As with low contrasts, the addition of an inhibitory input with balanced conductance, *μ_i_ = μ_e_*, shifts the rate/conductance curve down, reducing the maximum spike rate to ∼40 spikes/s with 100 μS/cm^2^ (Figure 2B,E). However, in the balanced current regime, *μ_i_ = 5μ_e_*, increasing the total mean conductance shifts the rate/conductance curve down, reducing the maximum spike rate to ∼25 spikes/s with 100 μS/cm^2^ (Figure 2B,F). Again, these downward shifts act as a divisive gain control, reducing sensitivity and increasing the range over which the compartment can operate. Thus, by adjusting the amount of inhibition it is possible to tune the responses of the post-synaptic neuron (Figure 2B, right panel). Comparing different contrast levels in the approximately balanced current regime shows that the curve relating spike rate to excitatory conductance becomes steeper at higher contrasts (Figure 2C). Thus, increasing the slope of the F–I curve is not only a property of the intrinsic biophysics but is also strongly dependent upon the input stimulus statistics (*cf.* Stemmler and Koch [21]; Figure 2).

### Information coding

**Figure 3:**
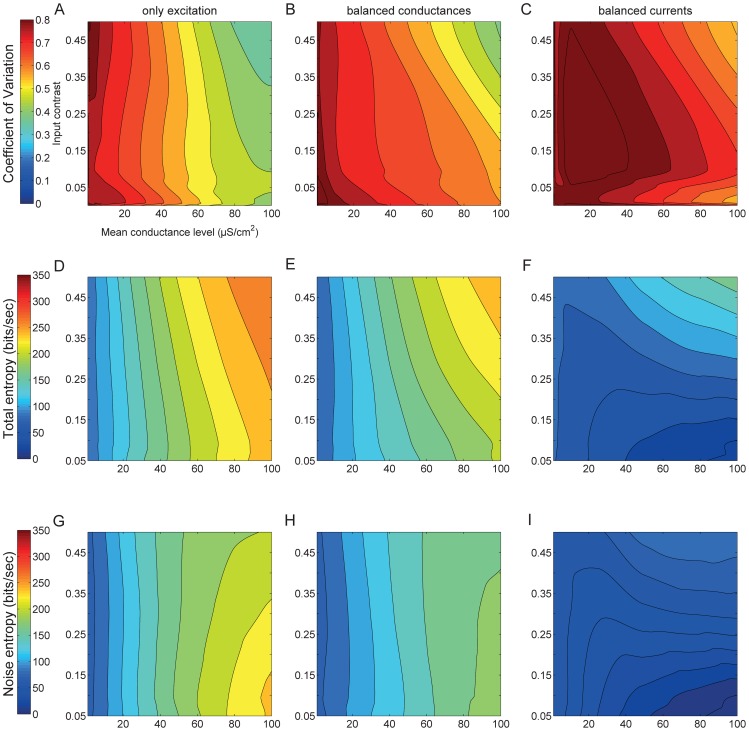
Irregularity and entropy of spike trains evoked by three different synaptic input regimes. (A) The Coefficient of Variation (CV) of the interspike interval distribution of spike trains generated by excitation alone. (B) As in A, except that an identical inhibitory synaptic input has been added. (C) As in A, except that the excitatory input is accompanied by a five-fold greater inhibitory synaptic input. (D) The total entropy of responses of spike trains generated by excitation alone. (E) As in D, except that an identical inhibitory synaptic input has been added. (F) As in D, except that the excitatory input is accompanied by a five-fold greater inhibitory synaptic input. (G) The noise entropy of responses of spike trains generated by excitation alone. (H) As in G, except that an identical inhibitory synaptic input has been added. (I) As in G, except that the excitatory input is accompanied by a five-fold greater inhibitory synaptic input. The x- and y-axes represent the mean and contrast of the excitatory conductance.

Differences in the inter-spike intervals of spikes evoked by the three synaptic regimes were quantified using the coefficient of variation (CV) (see Methods). Irrespective of the stimulus contrast, excitatory synaptic inputs alone generated spike trains with a high CV when the mean conductance was low (Figure 3A). The addition of inhibitory synaptic inputs of the same mean conductance and contrast increased the CV, indicating greater irregularity in the spike trains, even at high mean conductance levels (Figure 3B). Increasing the inhibitory synaptic inputs to balanced currents, *μ_i_ = 5μ_e_*, increased the CV still further, indicating even greater irregularity in the spike trains (Figure 3C).

The CV confounds variation due to the fluctuating synaptic input (signal) with noise generated by the stochastic activation of voltage-gated Na^+^ and K^+^ channels. Noise is identified by comparing responses to repeated presentations of the same signal and its effects on coding accounted for with information theoretic metrics [22]. For a given stimulus, the total entropy is a measure of the repertoire of spiking patterns that can be produced by the compartment, setting its information capacity [23]. We measured the total entropy by presenting a different conductance waveform on each subsequent trial (unfrozen noise) (see Methods). The total spike train entropy generated by excitatory synaptic inputs alone increases with the mean conductance, *μ_e_* (Figure 3D). The addition of inhibitory synaptic inputs with the same mean and contrast decreases the total entropy (Figure 3E), and entropy decreases still further when the current is approximately balanced by increasing the inhibitory input so that *μ_i_ = 5μ_e_* (Figure 3F).

We also presented the same conductance waveform repeatedly (frozen noise) to quantify the noise entropy of the responses (see Methods), which is a measure of spike train reproducibility [23]. With purely excitatory inputs of low contrast the noise entropy increases with mean conductance (Figure 3G). The addition of inhibition that balances the excitatory conductance, *μ_i_ = μ_e_*, decreases the noise entropy (Figure 3H *cf.* Figure 3G), and again noise entropy increases as synaptic conductance increases. Increasing the relative strength of inhibition to approximately balance current, *μ_i_ = 5μ_e_*, greatly reduces noise at all combinations of contrast and mean conductance (Figure 3I), making the spikes more reproducible from trial to trial.

**Figure 4:**
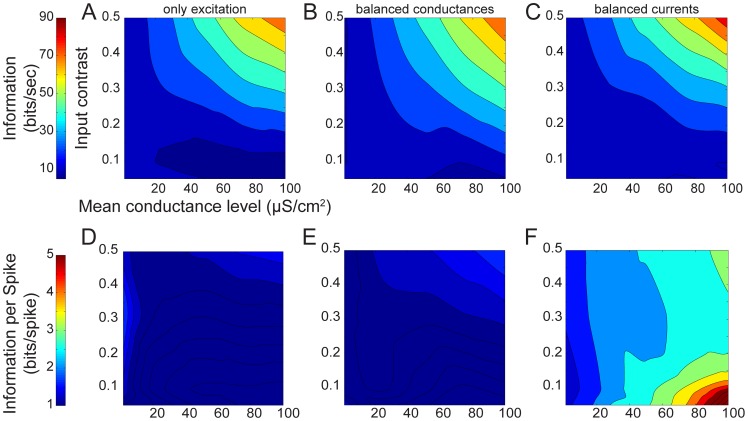
Information rate and coding efficiency evoked by three different synaptic input regimes. (A) The information rates of spike trains generated by excitatory conductances alone. (B) As in A but with identical excitatory and inhibitory conductances. (C) As in B but the inhibitory conductance is five-fold greater than the excitatory conductance in both mean and standard deviation. (D) The information per spike of spike trains generated by excitatory conductances alone. (E) As in D but with identical excitatory and inhibitory conductances. (F) As in E but the inhibitory conductance is five-fold greater than the excitatory conductance in both mean and standard deviation. The x- and y-axes represent the mean and contrast of the excitatory conductance.

The difference between the total and noise entropies determines the mutual information (MI) of the spike trains, a direct measure of the amount of information free of assumptions about how the information is represented and what it means [23]. We calculated the MI represented in the spike trains generated by each synaptic input regime (Figure 4A–C). The information rate increases with input contrast when the synaptic inputs are purely excitatory (Figure 4A) because increasing contrast increases the signal amplitude, and hence the signal-to-noise ratio (SNR) within the model compartment. Incorporating inhibition identical to the excitation (Figure 4B) had little effect on the information rates, and they vary with contrast and mean conductance level in the same way. The changes are small because the addition of inhibition reduces the total entropy and the noise entropy by equivalent amounts (median reduction is 1.1 fold). When inhibition is increased to approximately balance currents, *μ_i_ = 5μ_e_*, the noise entropy reduces by a factor of 2.3 fold (averaged across the range of contrasts and mean conductance levels) and the total entropy reduces 1.7 fold (Figure 4C). In other words, increased inhibition produces highly irregular spike trains that are precisely timed over trials. This simultaneous yet unequal drop in both total entropy and noise entropy produces a marginally better information encoding – the area of poor encoding (low bit rate) increases but there is a steeper rise to a higher bit rate at the highest values of contrast and mean conductance. Hence, more inhibition (approximately balanced currents) causes weak signals to perform worse and stronger signals to perform marginally better.

### Coding efficiency

Differences in the information rates of spike trains generated by the three synaptic regimes are dependent partly upon the spike rate (Figure 2D–F) [24]. However, by dividing the information rate by the corresponding spike rate for each conductance stimulus for a particular synaptic regime it is possible to determine the information encoded by each spike, the coding efficiency (Figure 4D–F). The coding efficiency of spikes evoked by excitation alone or by identical excitation and inhibition was similar; both attained between 0.1 and 2.4 bits/spike with the higher values being generated by high contrast, low mean stimuli (Figure 4D,E). Increased inhibition not only increases the coding efficiency across the entire stimulus space but also alters the trends so that low mean, low contrast stimuli evoke the most bits/spike (Figure 4F). The higher coding efficiency of the increased inhibition stimuli derives from the increased reliability and precision of the spikes they generate (Figure 3I) and emphasizes that although they achieve similar information rates to the other synaptic regimes, they do so despite far lower spike rates.

### Energy consumption

**Figure 5:**
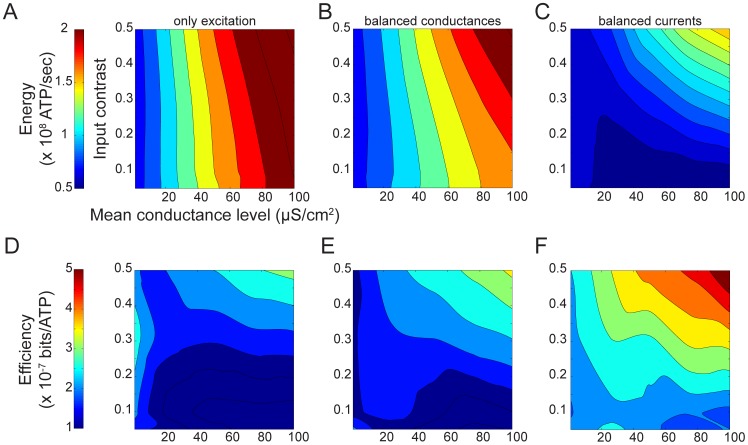
Energy consumption and energy efficiency evoked by three different synaptic input regimes. (A) The energy consumption of spike trains generated by excitatory conductances alone. (B) As in A but with identical excitatory and inhibitory conductances. (C) As in B but the inhibitory conductance is five-fold greater than the excitatory conductance in both mean and standard deviation. (D) The energy efficiency of spike trains generated by excitatory conductances alone. (E) As in D but with identical excitatory and inhibitory conductances. (F) As in E but the inhibitory conductance is five-fold greater than the excitatory conductance in both mean and standard deviation. The x- and y-axes represent the mean and contrast of the excitatory conductance.

The ion fluxes across the membrane that generate electrical signals and noise in neurons consume energy because the Na^+^/K^+^ ATPase must expel Na^+^ ions and import K^+^ ions against their concentration gradients, using the energy provided by ATP [16], [25], [26]. The ATPase hydrolyzes one ATP molecule to ADP to expel 3 Na^+^ ions and import 2 K^+^ ions and this stoichiometry allows one to calculate the energy consumption (Methods) from the total fluxes of Na^+^ and K^+^ across the membrane [16]. In all three synaptic regimes, the model's energy consumption increased with the excitatory synaptic conductance so that spike trains generated by high mean, high contrast stimuli used the most energy (Figure 5A–C). Comparison among the three synaptic input regimes shows that energy consumption across the entire stimulus space drops as inhibition increases (Figure 5A–C).

The total energy consumption of our single compartment model is determined by the currents flowing through the excitatory and inhibitory synaptic conductances, the voltage-gated ion channels that generate the action potentials, and the leak conductance [16], [27]. We partitioned the energy consumption into these component parts to determine their relative contributions (see Methods) (Figure S1). Under all synaptic regimes, and with all combinations of contrast and mean synaptic conductance, the currents flowing through voltage-gated ion channels during action potentials (Figure S1A–I) were the primary energy consumers. This explains why the trends in energy consumption (Figure 2D–F, 5A–C) closely resemble those in spike rate (Figure 2E–F).

In both the excitation only and balanced conductance regimes, action potentials account for between 85 and 90% of the total energy consumption, and the highest AP consumption occurring with high mean, high contrast stimuli (Figure S1C,F). The majority of the remaining energy is consumed by the leak conductance, between 5–12%, the energy consumed decreasing as the stimulus mean increases (Figure S1A,D). The synaptic currents account for just 2–4% of the total energy consumption, increasing with higher stimulus means (Figure S1B,E).

Increasing inhibition to approximately balance the excitatory synaptic current, *μ_i_ = 5μ_e_*, reduces the fraction of the energy consumed by the voltage-activated currents to between 50 and 80% (Figure S1I). These active currents consume the least energy with high mean, low contrast stimuli, the costs rising with increasing contrast or decreasing stimulus mean (Figure S1I). The opposite trend occurs for the synaptic costs, which rise from 2 to 30% of the total energy consumption as the stimulus mean increases and the contrast decreases (Figure S1H). The leak current consumes between 7 and 15%, the highest consumption occurring at low contrasts (Figure S1). These trends can be explained by the reduced spike rates evoked by low contrast stimuli, especially with high mean stimuli, which cause the spike rate to drop below the spontaneous spike rate (Figure 2F).

### Energy efficiency

The energy efficiency (bits/ATP molecule) of a spike train can be calculated by dividing the mutual information rate (bits/s) by the energy consumed (ATP molecules/s). Increased inhibition generates spike trains that are more efficient than either excitation alone or identical excitation and inhibition irrespective of the stimulus mean and contrast (Figure 5D–F). Increasing both the mean and the contrast of the stimulus produces the highest energy efficiency, up to 5*10^−7^ bits/ATP molecule for increased inhibition (Figure 5D–F) attributable to a drop in spike rate, which reduces total consumption while coding efficiency, the number of bits carried by each spike, increases (Figure 5D–F).

### Net currents and efficiency

**Figure 6:**
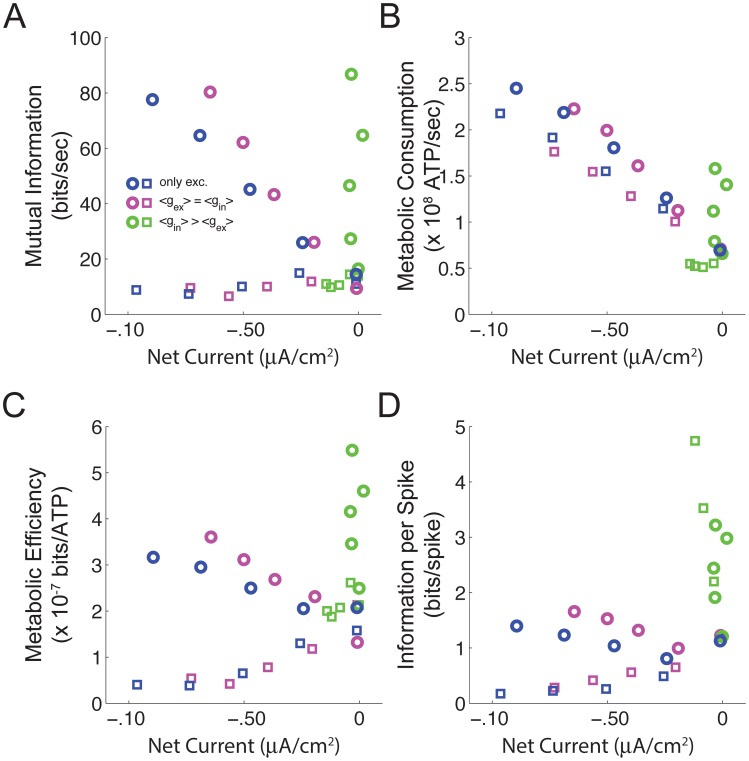
Approximate balance of excitation and inhibition. (A) The mutual information of spike trains from all three synaptic regimes with high (circles) and low contrast (squares) stimuli *versus* the net current. (B) As in A but for the energy consumption. (C) As in A but for energy efficiency. (D) As in A but for the coding efficiency. Open squares indicate a low input contrast (0.05). Open circles indicate a high input contrast (0.5). Data are re-plotted from Figures 4 and 5.

We compared the performance of the three synaptic regimes in terms of the net currents that they produce with low and high contrast stimuli. Both the excitation alone and the equal excitation and inhibition regimes generated an increasingly large net inward current as the mean excitatory synaptic conductance increases, irrespective of the stimulus contrast (Figure 6). However, when the inhibitory conductance is five-fold greater than the excitatory, there is no net current flow (Figure 6). Comparison of the three regimes shows that balanced synaptic currents generate spike trains with higher mutual information rates (Figure 6A), and lower energy consumption (Figure 6B) than either of the regimes that produce a higher net current. Because of the low spike rates generated by balanced synaptic currents, this results in improved metabolic efficiency (Figure 6C), and more information per spike (Figure 6D) than the other synaptic input regimes.

## Discussion

We have shown that approximately balanced inhibitory and excitatory synaptic currents increase both coding efficiency and energy efficiency in comparison to two other synaptic input regimes – excitation alone, and balanced excitatory and inhibitory conductances. Key to this improvement in efficiency is a reduction in spike rate and an increase in spike timing precision. The strong inhibitory conductance needed to generate a current that balances the excitatory current produced the lowest spike rates of all the regimes we studied across the entire input stimulus space. This reduction in spike rate is responsible for an overall drop in energy consumption (ATP molecules/s) because the voltage-gated currents that generate APs dominate the energy consumption of all the models. In the balanced synaptic current regime, the energy savings from lower spike rates are sufficient to offset the increased costs of the synaptic conductances. Yet, despite generating fewer spikes, the information rates of spike trains generated by balanced synaptic currents in our models are similar to those generated by excitation alone or by balanced excitatory and inhibitory conductances. Thus, balanced synaptic currents increase coding efficiency (bits/spike) rather than the information rate (bits/s). By reducing energy consumption and increasing coding efficiency, approximately balanced synaptic currents increase the energy efficiency (bits/ATP molecule) of spike trains compared to the other synaptic regimes we modeled.

Our conclusions are based upon comparisons among single compartment models that incorporated a well-established model of synaptic input that accounts for the Poisson distribution of spikes in cortical neurons [20]. The model assumes that large numbers of weak synapses are activated individually by afferent spikes that, because they come from a large population of neurons firing with Poisson statistics, are largely uncorrelated [28]. Excitatory and inhibitory synaptic inputs to our models were uncorrelated, noise free, and had identical synaptic time constants. However, within cortical networks excitation and inhibition are continuously synchronized and correlated in strength [29]. Even small differences in the timing of excitation and inhibition can modulate neuronal integration time, forming a selective gate for signal transients that affects information processing [30]. The addition of noise to the time-varying sub-threshold synaptic input of a spiking neuron can increase the regularity (periodicity) of spiking (stochastic resonance) [31] or, in the absence of time-varying input, additive noise can lead to patterned firing (coherence resonance) [32]. In the absence of these effects, the addition of noise to synaptic currents will degrade the quality of the input signal (SNR), decreasing the information rate through an increase in noise entropy [33]. However, noise in the inhibitory and excitatory conductances will be multiplicative rather than additive with consequences for post-synaptic firing rates, information coding and metabolic efficiency [34]–[36].

Post-synaptic inhibitory conductance changes can be phasic or tonic [37]; phasic inhibition supports rhythmic activity in neuronal networks, such as the theta or the gamma oscillations [38], [39], whereas tonic inhibition increases conductance affecting signal integration. These specific characteristics have consequences for their effects on neuronal gain control. For example, blockage of tonic inhibition can shift the input-output relationship of cerebellar granule cells to the left (subtractive gain control) depending upon the temporal properties of the excitatory conductance [40], [41]. Random trains of excitatory conductance cause a divisive as well as a subtractive modulation of gain [42]. Although our models encompass a limited set of excitatory and tonic inhibitory input properties that capture qualitatively similar modulation of neuronal gain to that observed empirically, cortical circuits incorporate numerous other combinations of phasic/tonic inhibition and static/random trains of excitation that can modulate gain and affect information processing.

We use the simplest possible model of synaptic integration, an electrotonically compact compartment in which synaptic inputs directly drive a membrane containing the minimum set of voltage-gated conductances [43]. Consequently, our models do not account for the complex structure of pyramidal neurons [1], [3] and the spatial distribution of excitatory and inhibitory inputs [44]–[46]; excitatory inputs are formed mainly on dendritic spines, whereas inhibitory synapses are located primarily on dendritic shafts, the soma and the axon initial segment [47]. Synaptic inputs are shaped and filtered by both passive membrane properties and active conductances in pyramidal neurons [48] that will affect both information processing and energy consumption.

The voltage-gated ion channel properties in our models are taken from the squid giant axon because detailed kinetic models exist for these voltage-gated channels [49]. However, the squid action potential is profligate in its energy usage, consuming ∼17-fold more energy than some vertebrate action potentials [27], suggesting that channels with different kinetics will reduce energy consumption [17]. Our calculations of energy consumption also do not incorporate the presynaptic cost of generating the synaptic conductances. Inhibitory neurons typically have higher firing rates and form more, stronger synaptic connections than excitatory neurons [50]. However, in the cortex, inhibitory neurons are smaller and less numerous than excitatory neurons [2]. This suggests that the pre-synaptic cost of generating inhibitory conductances is lower than generating excitatory conductances and, indeed, cortical energy budgets have ignored the cost of inhibition entirely [16], [27].

Yet because our analysis is basic, it reveals some biophysical principles of efficient coding. In our model, balanced inhibitory and excitatory currents increase coding efficiency by reducing the number of action potentials and increasing their spike timing precision in the face of channel noise. This sparsening of the output spike train is due to the strong inhibitory conductance needed to generate a current that balances the excitatory current. Sparser codes translate into fewer spikes or the activation of fewer neurons in a network, reducing redundancy [51]. Our work shows that such temporal sparseness [52] is produced by an increase in inhibition that makes the neuron more efficient by increasing the information (bits) per spike. A reduction in spike rate also tends to increase the information per spike because spikes become more surprising [24]. Increased spike timing precision is a consequence of a faster membrane time constant and larger changes in conductance ratios creating a faster-rising voltage slope, which again increases the bits per spike. Neurons may reach high firing rates, thereby incurring a heavy metabolic cost, but they can do so only momentarily. Thus, our model demonstrates that inhibition can improve efficiency by facilitating efficient sparser codes by acting on fundamental determinants of coding efficiency.

By increasing the information per spike and reducing the spike rate balanced synaptic currents maximize information rate within a limited energy budget. This is particularly important when considered in the context of cortical energy budgets, which limit average firing rates to ∼7 Hz [27] in rat grey matter and probably to even lower rates in humans. Fewer, more informative action potentials can save energy not just in a single neuron but throughout the cortical network [18], [53], by ensuring that synapses activate only to transmit signals from more informative spikes, thereby increasing their efficiency with which they are used. A single cortical neuron makes recurrent excitatory synaptic connections with many other cortical neurons, of which about 85% of the synapses are with other excitatory neurons [2], [3], [55], [56]. Despite these synaptic connections being weak [1], spiking activity can propagate through cortical networks [57]. Indeed, even a single additional spike can lead to a large number of extra spikes in downstream neurons [58]. Thus, even small changes in spike rate can inflate energy costs by evoking additional spikes in post-synaptic neurons.

The role of balanced synaptic currents appears to be to allow cortical neurons to process information with low numbers of precise spikes. This is only possible if neurons have fast membrane time constants, sit close to the spike initiation threshold, and depolarize rapidly to conductance changes to produce spikes. These features inflate energy costs suggesting that a low cost resting state that is separated from a high cost ‘active’ state would be advantageous. It seems likely that the cortex has been under considerable pressure to reduce energy consumption whilst retaining the ability to respond rapidly and precisely. Balanced inhibition/excitation appears to be an answer to this problem. When not in ‘active’ use, cortical neurons can sit far from rest with slow membrane time constants incurring relatively low energy costs. When active the balanced synaptic currents depolarize and speed up the cortical neurons allowing them to respond rapidly to synaptic inputs. Thus, balanced synaptic currents effectively uncouple resting and active states in terms of energy use, saving energy when neurons are at rest.

We have made a basic general model that reveals that current balanced excitation and inhibition can increase coding efficiency, improving the statistics of spike trains by increasing signal entropy and reducing noise entropy. Energy efficiency also improves due to a reduction in spike rate. This suggests that despite their extra cost, inhibitory synapses will increase the energy efficiency of circuits performing a wide variety of functions by making spikes more informative.

## Materials and Methods

### Single compartment model with conductance noise

We simulated single compartment models containing stochastic voltage-gated ion channels, the properties of which were based on the original Hodgkin-Huxley model of a squid axon [49], [59]. The model contained transient voltage-gated Na^+^ channels and delayed rectifier voltage-gated K^+^ channels along with a non-probabilistic voltage independent leak conductance. The dynamics of the membrane potential was governed by the following current balance equation:
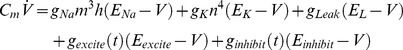
(1)where C_m_ is the membrane capacitance, g_Na_, g_K_ and g_Leak_ are the conductances of the Na^+^, K^+^ and leak channels, respectively. E_j_ are the reversal potentials of these conductances, where 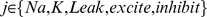. The variables m, h and n follow first order kinetics of the form 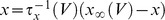, where 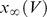 is the steady-state activation or inactivation function and 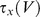 is the voltage-dependent time constant. The single compartment model is driven either by an excitatory conductance, 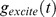, or in addition to an inhibitory conductance, 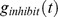. The exact forms of conductance fluctuation that give rise to the synaptic currents are described in the next section. In our simulations the synaptic conductances were modeled to be noise-free.

### Diffusion approximations

We model the source of the synaptic conductance as a large number of weak synaptic inputs, each driven randomly and independently, as if by spikes from one unique neuron. This diffusion approximation [20] delivers a white noise synaptic current when the post-synaptic response is instantaneous, and pink noise when the post-synaptic response lasts for a finite time. For the diffusion approximation, we used the conductance model of Destexhe *et al*. [20] and define the synaptic conductances as,
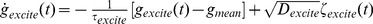
(2)where 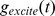 is the time-dependent excitatory conductance, 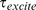 is the time constant that defines the decay time of the synaptic activation in response to Poisson distributed spike trains, and 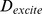 is the diffusion coefficient of the noise process while 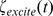 is a zero mean and unit standard deviation Gaussian noise process. 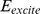 was set to 0 mV and 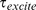 was fixed at 3.3 ms. The inhibitory conductance trace was generated by an identical yet independent differential equation, differing only in the choice of 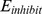 which was set to −75 mV.

The conductances were modeled as an Ornstein-Uhlenbeck (OU) process. The OU process is a model for a large number of randomly activated synaptic inputs impinging on the single compartment, where each input is simply approximated using a single exponentially decaying conductance. The conductances generated using an OU process have approximately a Gaussian distribution with a Lorentzian power spectrum. Because of this Gaussian distribution, the differential equation can be written as a difference equation, which is independent of step size Δ,
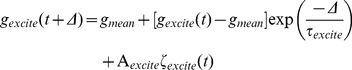
(3)

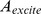
 is the amplitude of the noise such that,
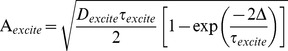
(4)The mean synaptic conductance *μ* (in Siemens), depends upon the rate, *R* Hz, the unitary synaptic event amplitude, *A* Siemens, and the exponential decay time constant *τ* (in seconds) of synaptic events
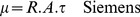
(5)the standard deviation of synaptic conductance, *σ*, is given by
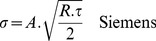
(6)and the conductance contrast, is
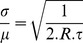
(7)Note that the stimulus contrast only depends on the event rate, *R*, and the decay time constant, *τ*, which in this study is fixed. Thus, when we increase the conductance contrast we are decreasing the event rate (i.e. reducing the frequency with which afferent spikes activate synapses), and when we increase the mean conductance at constant contrast we are increasing event amplitude at constant rate.

The stimulus was presented for 1 second and each set of simulations consisted of 60 such trials. All Gaussian random numbers were generated using the Marsaglia's Ziggurat algorithm; uniform random numbers were generated using Mersenne Twister algorithm. Deterministic equations were integrated using the Euler-algorithm while stochastic differential equations were integrated using the Euler-Maruyama method; both with a step size of 10 µs. Parameter values are given in Table S1. Markov state transitions for the voltage-gated ion channels are modeled after the channel noise formulation in Refs. [49], [60].

### Calculating information rates

We used the “direct method” to measure the entropy of the responses [61], [62], which compares different spike trains without reference to the stimulus parameters. The total entropy sets the information capacity for the spike train. The noise entropy measures the variability of the spike train across trials. These quantities were dependent upon the temporal resolution with which the spikes were sampled, 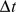 (1 ms), and the size of time window, *T*. We presented either a different conductance trace in each subsequent trial (unfrozen noise) to calculate the total entropy, or the same conductance trace in each subsequent trial (frozen noise) to calculate the noise entropy. We divided the spike train to form K-letter words (K = 2, 4, 6, 8, 12, 16, 24, 32, 48 or 64), where 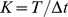. We used the responses from the unfrozen noise presentations (60 trials each of 1 second) to estimate the probability of occurrence of particular word, 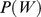. The total entropy was estimated as,
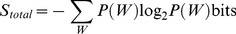
(8)


We estimated the probability distribution of each word at the beginning of each work at time *t* to obtain the conditional probability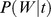. Entropy estimates were then calculated from these distributions and the average of the distributions at the different starting times *t* was computed to give the noise entropy (60 trials each of 1 second) as,
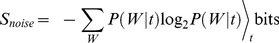
(9)where, 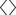 indicates average over time. The mutual information was then computed as,
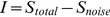
(10)The total entropy and the conditional noise entropy diverge in the limit 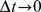, their difference converges to the true finite information rate in this limit 61,62]. Therefore, we used bias correction methods to reduce the effect of sampling errors [63]. Using 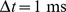, we varied the spike trains to form words of different lengths. Using these entropy estimates, we extrapolated to infinite word length from the four most linear values of the plot of entropy and the inverse word length.

### Calculating energy consumption

The energy consumption of each compartmental model is determined by the number of ATP molecules expended per second, averaged over 60 trials of 1 second each. The Na^+^/K^+^ pump hydrolyses one ATP molecule for three Na^+^ ions extruded and two K^+^ ions imported [26], [64]. Assuming that the two main charge carriers in a cell are due to Na^+^ and K^+^ we divided the excitatory, inhibitory and leak conductances into separate pools of Na^+^/K^+^ permeable conductances. We then determined the total K^+^ permeable current and added it to the delayed rectifier K^+^ current. We computed the number of K^+^ ions by integrating the area under the total K^+^ current curve for the duration of stimulus presentation. Finally, we calculated the number of ATP molecules used by multiplying the total K^+^ charge by 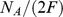, where 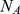 is Avogadro's constant and *F* is Faraday's constant. Pre-synaptic costs (transmitting an AP to the pre-synaptic terminal, transmitter release and recycling) are not included in our analysis. The presynaptic costs of calcium entry and transmitter release and recycling are approximately one fifth the cost of post-synaptic current [16], [65].

## Supporting Information

Figure S1 **The composition of metabolic consumption of spike trains evoked by three different synaptic input regimes.** Left column: Contribution of the leak current to the total metabolic consumption. Middle column: Contribution of the synaptic current to the total metabolic consumption. Right column: Contribution of the active current to the total metabolic consumption. A–C only excitation. D–F Excitation and inhibition. G–I More inhibition. The x- and y-axes represent the mean and contrast of the excitatory conductance.
(TIF)

Table S1 **Parameters for the stochastic Hodgkin-Huxley model.**
(DOCX)
