# Supplementary figures and images for: Balanced Excitatory and Inhibitory Synaptic Currents Promote Efficient Coding and Metabolic Efficiency
Source: PLoS Comput Biol. 2013 Oct 3;9(10):e1003263. doi: 10.1371/journal.pcbi.1003263 (PMC3789774; doi:10.1371/journal.pcbi.1003263)

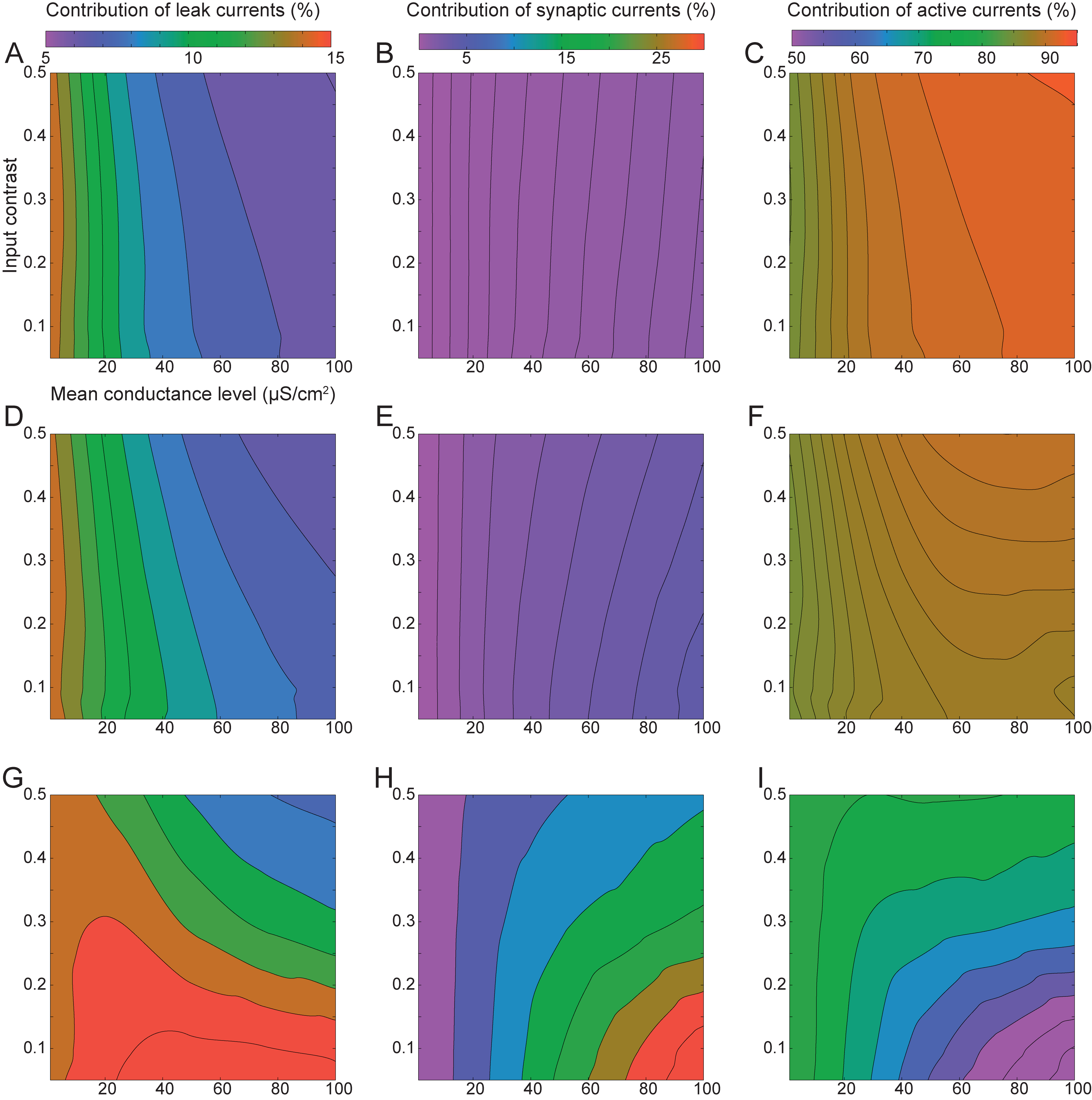

Supplement: Figure S1 — The composition of metabolic consumption of spike trains evoked by three different synaptic input regimes. Left column: Contribution of the leak current to the total metabolic consumption. Middle column: Contribution of the synaptic current to the total metabolic consumption. Right column: Contribution of the active current to the total metabolic consumption. A–C only excitation. D–F Excitation and inhibition. G–I More inhibition. The x- and y-axes represent the mean and contrast of the excitatory conductance. (TIF) [file pcbi.1003263.s001.tif]
