# Supplementary material for: Balanced Excitatory and Inhibitory Synaptic Currents Promote Efficient Coding and Metabolic Efficiency
Source: PLoS Comput Biol. 2013 Oct 3;9(10):e1003263. doi: 10.1371/journal.pcbi.1003263 (PMC3789774; doi:10.1371/journal.pcbi.1003263)
Supplement: Table S1 — Parameters for the stochastic Hodgkin-Huxley model. (DOCX) [file pcbi.1003263.s002.docx]

**Table S1 Parameters for the stochastic Hodgkin-Huxley model**

| SYMBOL | DEFINITION | VALUE, UNITS |
| --- | --- | --- |
| C_m_ | Specific membrane capacitance | $1\text{ }\mu F/cm^{2}$ |
| T | Temperature | $6.3\text{ }^{\circ}C$ |
| E_L_ | Leakage reversal potential | $-54.4mV$ |
| E_Na_ | Sodium reversal potential | $50\text{ }mV$ |
| E_K_ | Potassium reversal potential | $-77mV$ |
| g_Leak_ | Leakage conductance | $0.3\text{ }mS/cm^{2}$ |
| N_Na_ | Na^+^ channel density | $60/\mu m^{2}$ |
| N_K_ | K^+^ channel density | $18/\mu m^{2}$ |
| $\alpha_{m}(V)$ | Opening rate (activation, Na^+^) | $\frac{0.1(V+40)}{1-\exp\left(-0.1(V+40)\right)}$ |
| $\alpha_{h}(V)$ | Opening rate (inactivation, Na^+^) | $0.07\exp\left(-0.05\left(V+65\right)\right)$ |
| $\alpha_{n}(V)$ | Opening rate (activation, K^+^) | $\frac{0.01(V+55)}{1-\exp\left(-0.1(V+55)\right)}$ |
| $\beta_{m}(V)$ | Closing rate (activation, Na^+^) | $4\exp\left(-0.0556\left(V+65\right)\right)$ |
| $\beta_{h}(V)$ | Closing rate (inactivation, Na^+^) | $\frac{1}{1+\exp\left(-0.1(V+35)\right)}$ |
| $\beta_{n}(V)$ | Closing rate (activation, K^+^) | $0.125\exp\left(-0.0125\left(V+65\right)\right)$ |
